# Overexpression of *MsDREB1C* Modulates Growth and Improves Forage Quality in Tetraploid Alfalfa (*Medicago sativa* L.)

**DOI:** 10.3390/plants13091237

**Published:** 2024-04-29

**Authors:** Yangyang Zhang, Zhen Wang, Fan Zhang, Xue Wang, Yajing Li, Ruicai Long, Mingna Li, Xianyang Li, Quanzhen Wang, Qingchuan Yang, Junmei Kang

**Affiliations:** 1 Institute of Animal Science, Chinese Academy of Agricultural Sciences, Beijing 100193, China; yang20170115@163.com (Y.Z.); 15600085453@163.com (F.Z.); wangxue01@caas.cn (X.W.); ly_yajing@yeah.net (Y.L.); longruicai@caas.cn (R.L.); limingna@caas.cn (M.L.); xianyangli2022@163.com (X.L.); 2 College of Grassland Agriculture, Northwest A&F University, Yangling 712100, China; 3 Department of Agronomy and Horticulture, University of Nebraska-Lincoln, Lincoln, NE 68583, USA; zwunl3@gmail.com

**Keywords:** abiotic stress, alfalfa, overexpression of *MsDREB1C*, growth retardation, GA, lignin content

## Abstract

DREB has been reported to be involved in plant growth and response to environmental factors. However, the function of *DREB* in growth and development has not been elucidated in alfalfa (*Medicago sativa* L.), a perennial tetraploid forage cultivated worldwide. In this study, an ortholog of *MtDREB1C* was characterized from alfalfa and named *MsDREB1C* accordingly. *MsDREB1C* was significantly induced by abiotic stress. The transcription factor MsDREB1C resided in the nucleus and had self-transactivation activity. The *MsDREB1C* overexpression (OE) alfalfa displayed growth retardation under both long-day and short-day conditions, which was supported by decreased *MsGA20ox* and upregulated *MsGA2ox* in the OE lines. Consistently, a decrease in active gibberellin (GA) was detected, suggesting a negative effect of *MsDREB1C* on GA accumulation in alfalfa. Interestingly, the forage quality of the OE lines was better than that of WT lines, with higher crude protein and lower lignin content, which was supported by an increase in the leaf–stem ratio (LSR) and repression of several lignin-synthesis genes (*MsNST*, *MsPAL1*, *MsC4H*, and *Ms4CL*). Therefore, this study revealed the effects of *MsDREB1C* overexpression on growth and forage quality via modifying GA accumulation and lignin synthesis, respectively. Our findings provide a valuable candidate for improving the critical agronomic traits of alfalfa, such as overwintering and feeding value of the forage.

## 1. Introduction

With the continuous growth of the global population, it is very important to ensure the sustainable development of global agriculture through breeding and genetic engineering [1]. Alfalfa is a superior-quality leguminous forage crop due to its high yield, good quality, and biological nitrogen fixation, and it has a decisive position in the technical system of animal husbandry industry [2]. Alfalfa is widely cultivated throughout the world, with 8.7 million hectares cultivated in the USA, making it the fourth-largest cultivated crop, after corn, soybean, and wheat [3,4,5]. In China, the planting area of alfalfa in 2015 was about 4.72 million hectares [6], making it the nation’s most widely cultivated perennial forage.

The content of crude protein and cellulose is the main evaluation indicator of forage quality in alfalfa [7]. The crude protein in alfalfa leaves accounts for a high proportion (260–300 g kg^−1^ DB) in the whole plant, which is an important source of forage protein [8]. However, the cellulose content of alfalfa is concentrated in its stems. Therefore, to ensure alfalfa yield, screening germplasms with a high leaf–stem ratio (LSR) and crude protein content is crucial to improve forage quality and reduce fertilizer application in order to promote sustainable agricultural development. In recent years, evidence has shown that by regulating the key synthetic genes of cellulose, such as *caffeoyl CoA 3-Omethyltransferase* (*CCoAOMT*) [9,10,11] and *NAC SECONDARY WALL THICKENING PROMOTING FACTOR* (*NST*) [12], the cellulose content of alfalfa can be reduced, thus laying the foundation for low-cellulose alfalfa breeding. In addition, studies have also shown that regulating the flowering time by regulating the expression of the *MsFT* gene can also improve the quality and yield of alfalfa [2,13]. However, due to the characteristics of self-incompatibly cross-pollinated autotetraploid and complex genetic information [3] of alfalfa, molecular breeding related to alfalfa quality improvement has been more difficult and slower than that of model crops.

The dehydration-responsive element-binding protein (DREB), also known as CBF (C-repeat binding transcription factor), a transcription factor of the APETALA2/ethylene-responsive element binding factor (AP2/ERF) family, has been implicated in plant growth and response to diverse environmental factors [14,15,16,17]. DREB1/CBF is the main switch of cold stress regulation, and its dependent signaling pathway has been reported to play an important role in cold resistance in *Arabidopsis thaliana* [18], *Glycine max* [14], *Oryza sativa* [19], *Medicago truncatula* [20], *Solanum lycopersicum* [21], and other plants. However, there is evidence that *DREB* has pleiotropic effects on plant growth retardance and improving abiotic resistance [20,21,22]. Currently, *DREB1/CBFs* mediated in growth response in Arabidopsis have been reported to be associated with gibberellin (GA) [23], a vital phytohormone for multiple physiological processes, including seed germination, stem elongation, floral organ development, and fruit ripening [23,24]. In addition to the function of increasing stress resistance and affecting growth, a recent study of field trials in three locations has shown that rice *OsDREB1C* overexpression lines improved photosynthetic efficiency, nitrogen use efficiency, and crop yield by more than 30% [1], suggesting that *OsDREB1C* has good potential to increase crop yield and efficient utilization of resources. Accordingly, the biological functions of *DREB1/CBFs* in plants are diverse and complex, and many mechanisms still need to be further explored. However, there is scarce information on the biological function(s) of *DREB* in alfalfa.

In a previous study, the pleiotropic effects, including improved freezing resistance and reduced plant growth, were associated with *MtDREB1C* in *M. truncatula*, the closest model legume of alfalfa [20]. However, the function of *DREB1C* in growth and development has not been well identified. In this study, alfalfa *DREB1C* (*MsDREB1C*), an abiotic stress-response gene sharing an identity of 71.4% with *MtDREB1C*, was characterized based on the phenotypic abnormalities of the *MsDREB1C* overexpression alfalfa plants. Relative to the non-transgenic alfalfa, two independent *MsDREB1C* overexpression alfalfa lines displayed growth retardance regardless of the culture conditions we tested. Moreover, the negative role of *MsDREB1C* in plant growth is related to the reduction of active gibberellin. Moreover, the forage possessed a significantly higher content of crude protein and lower lignin. Our findings suggest that the transcription factor encoding gene *MsDREB1C* is likely to be involved in multiple biological processes in tetraploid alfalfa, and provide a potential candidate for balancing traits, such as forage quality, plant growth, and stress resistance through genetic improvement.

## 2. Results

### 2.1. Msa0891980 Encodes a DREB1C in Alfalfa

**Figure 1:**
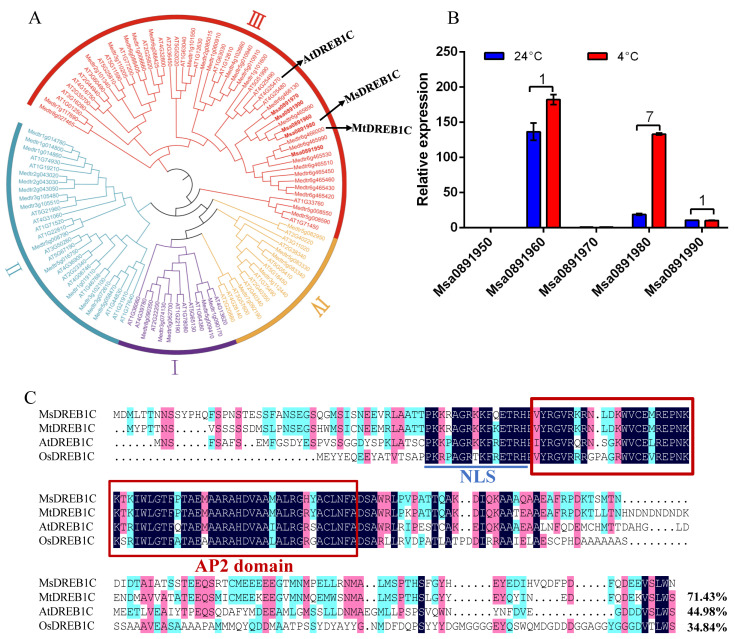
Homologous sequence alignment and phylogenetic analysis of MsDREB1C (Msa0891980). (**A**) Phylogenetic analysis of five MsDREB (Msa0891950, Msa0891960, Msa0891970, Msa0891980, and Msa0891990) and DREB family members from Arabidopsis and *M. truncatula*. Subgroups I–IV are indicated in color. (**B**) Expression analysis of the five putative *DREB* genes under cold treatment (4 °C) for 72 h in 4-week-old soil-cultured alfalfa. The numbers on the top of the column represent the fold change of cold treatment to the control conditions (24 °C). (**C**) Multisequence alignment of MsDREB1C with its orthologs from *M. truncatula* (MtDREB1C, Medtr6g466000), Arabidopsis (AtDREB1C, AT4G25470), and *Oryza sativa* (OsDREB1C, BGIOSGA022219-PA). The AP2 domain is framed, and the nuclear localization signal (NLS) underlined. The percentage on the bottom represents the sequence homology of MsDREB1C to its orthologs.

Previous research on the dehydration-responsive element-binding protein/C-repeat binding transcription factor (DREB1/CBF) in *M. truncatula*, the closest model legume of alfalfa, has indicated that overexpression of *MtDREB1C* (*Medtr6g466000*) results in pleiotropic effects, including improvement of freezing resistance and reduction of plant growth [20]. Homology analysis showed that multiple alfalfa orthologues (e.g., *Msa0891950*-*Msa0891990*) residing on chromosome 6 in tandem were clustered into the same subfamily of *MtDREB1C* and *AtDREB1C* (Figure 1A and Table S1). Transcriptional analysis of the leaves from soil-cultured alfalfa seedlings that were 4 weeks old revealed that two (*Msa0891950* & *Msa0891970*) of the five alfalfa paralogues were hardly detected by quantitative RT-PCR, and among the three expressed genes, *Msa0891980* was upregulated by 4 °C treatment for 72 h relative to the control conditions (Figure 1B). Thus, *Msa0891980* was named *MsDREB1C*.

The putative MsDREB1C consists of 229 amino acids with a molecular weight of 25.9 kDa and an isoelectric point (pI) of 5.55. Multiple sequence alignment showed that MsDREB1C shared a conserved AP2 domain with the DREB family members. Similar to its orthologues, MsDREB1C was predicted to possess a nuclear localization signal (NLS) (PKKRAGRKKFQETRHP) (Figure 1C). MsDREB1C shared a higher homology with MtDREB1C from *M. truncatula* (71.43%) relative to AtDREB1C from Arabidopsis (44.98%) and OsDREB1C from rice (34.84%) (Figure 1C).

### 2.2. MsDREB1C Responses to Multiple Abiotic Stresses

**Figure 2:**
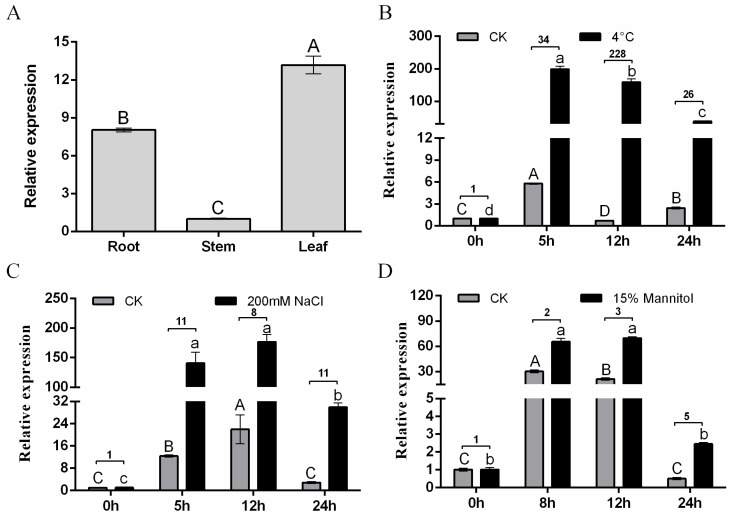
Expression analysis of *MsDREB1C* in alfalfa tissues under the indicated treatments. (**A**) *MsDREB1C* expression level in the tissues from 28-day-old alfalfa grown in Hoagland. Transcriptional analysis of *MsDREB1C* under 4 °C and control conditions (24 °C) (**B**), 200 mM NaCl (**C**), and 15% mannitol (**D**). For RNA extraction, leaves of 28-day-old alfalfa subjected separately to 4 °C, 200 mM NaCl, or 15% mannitol for the indicated time points were harvested from at least 4 plants. Notes: Results were analyzed using a one-way ANOVA test. Different uppercase letters or different lowercase letters represent significance less than 0.05. The number between the two histograms represents the fold change of treatment to the control. Data are means ± SEM of three biological replicates.

Based on quantitative RT-PCR, *MsDREB1C* was expressed in leaves, roots, and stems of the 4-week-old hydroponically cultured seedlings, the expression being relatively higher in leaves and lower in stems (Figure 2A). The short-term (24 h) abiotic stress treatment showed that relative to the non-treated alfalfa in hydroponics, the transcriptional level of *MsDREB1C* was induced by stress (Figure 2B–D). Upon exposure to 4 °C, *MsDREB1C* in the seedlings increased by cold stress relative to the non-treatment control (Figure 2B). Under NaCl (200 mM) treatment, *MsDREB1C* was significantly induced and sustained at a relatively stable level during the treatment (Figure 2C). For the drought treatment mimicked by 15% mannitol, the expression of *MsDREB1C* was up-regulated 2-, 3-, and 5-fold at 8 h, 12 h, and 24 h, respectively, relative to the control (Figure 2D). These results indicated that *MsDREB1C* responded to the short-term treatments of low temperature, salt, and drought, and the alfalfa seedlings seemed to be hypersensitive to the former stress compared to the latter.

### 2.3. Nuclear Protein MsDREB1C Has Self-Transactivation Activity

**Figure 3:**
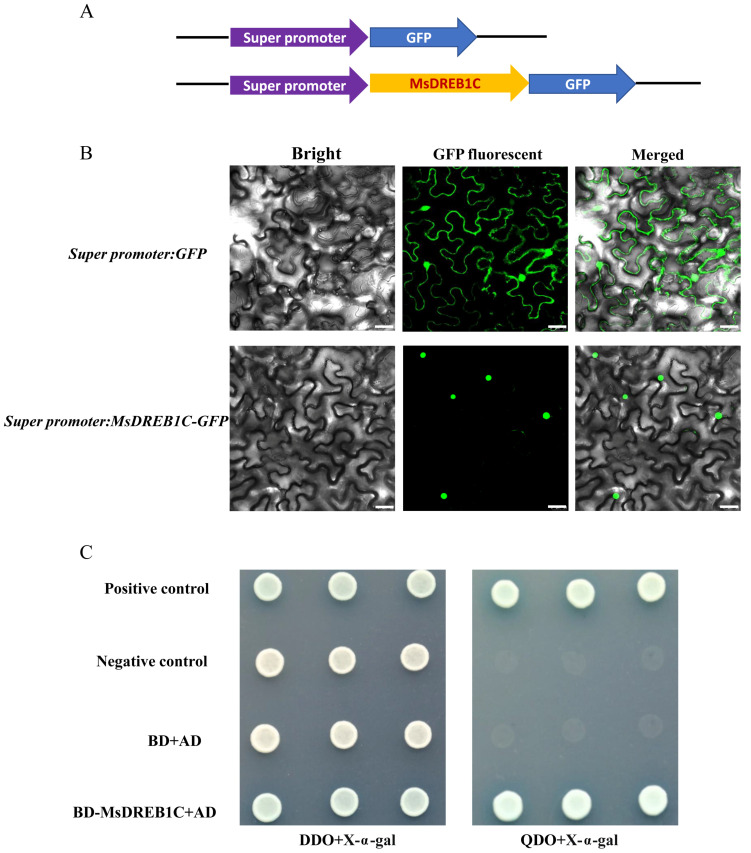
Analysis of subcellular localization and self-transactivation of MsDREB1C. (**A**) Schematic representation of the *Super promotor:GFP* and *Super promotor:MsDREB1C-GFP* fusion construct used in the fluorescence localization assay. (**B**) GFP and MsDREB1C-GFP fused proteins were expressed transiently in tobacco leaves by infiltration. Bar = 25 μm. (**C**) Self-transactivation assay of MsDREB1C in yeast. Yeast cells harboring the pGBKT7(BD)-MsDREB1C and pGBADT7(AD) co-transformation product were grown on two media. I: SD/-Trp, -Leu (DDO) medium with X-α-gal; and II: SD/-Trp, -Leu, -His, -Ade (QDO) medium with X-α-gal. The positive control was BD-53 and AD-T co-transformation product, and BD-lam and AD-T co-transformation product for the negative control. BD and AD co-transformation product was used as double negative control.

To test the prediction of a nuclear localization signal (PKKRAGRKKFQETRHP) in MsDREB1C (Figure 1C), *MsDREB1C* was constructed into a Super1300 expression vector to fuse with GFP (Figure 3A) and expressed transiently in tobacco leaf cells. As shown in Figure 3B, the GFP fluorescence signal of the control (*Super promotor:GFP* vector) was detectable throughout the epidermal cells of tobacco leaves, while the green fluorescence of the MsDREB1C-GFP recombinant protein was detected predominantly in the nucleus of leaf epidermal cells, confirming that MsDREB1C resides in the nucleus.

To assess whether the putative transcription factor MsDREB1C has self-transactivation activity, a pGBKT7(BD)-MsDREB1C fused vector was constructed. The yeast cells harboring BD-MsDREB1C and pGBADT7(AD) co-transformation product were cultivated on a control medium (DDO) of SD/-Trp, -Leu with X-α-gal, and a selection medium (QDO) of SD/-Trp, -Leu, -His, -Ade with X-α-gal. The results showed that in contrast to the control medium, on which all the tested cells grew happily on the selection medium, yeast cells harboring BD-MsDREB1C and AD co-transformation product, together with the positive control cells, grew normally (Figure 3C), indicating the self-transactivation ability of MsDREB1C.

### 2.4. Overexpression of MsDREB1C in Alfalfa Affects Plant Growth

**Figure 4:**
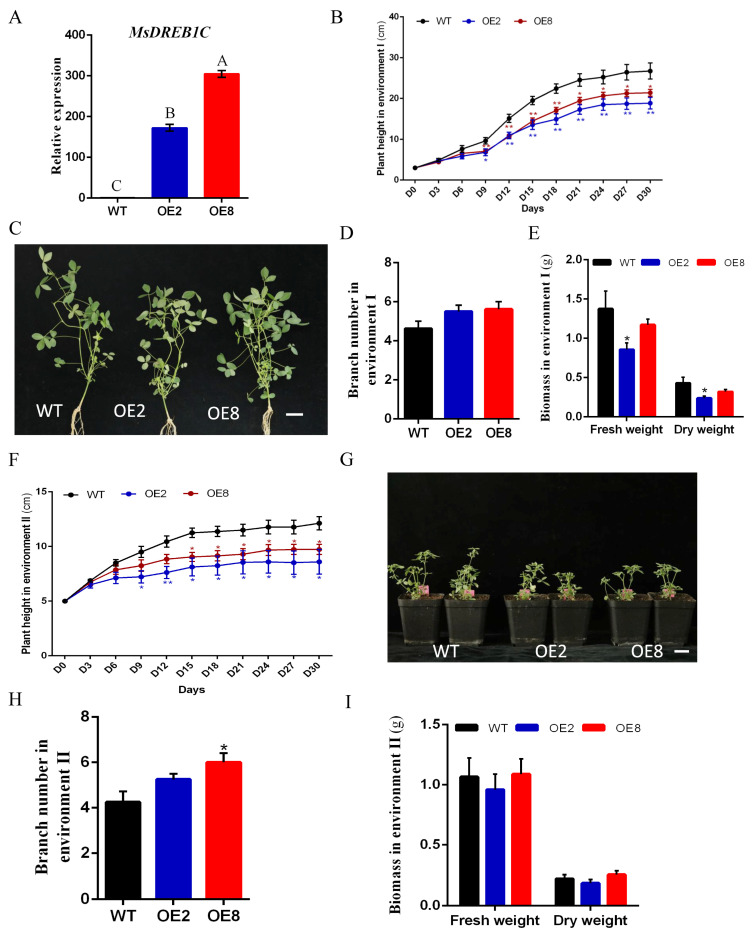
Overexpression of *MsDREB1C* in alfalfa affected plant growth. (**A**) Transcriptional analysis of *MsDREB1C* in the *MsDREB1C* overexpression alfalfa lines (OE2, OE8). (**B**) Plant height of the wild-type (WT) and transgenic plants (OE2, OE8) for 30 days in ‘environment I’, n = 8. ‘Environment I’ represents the conditions in the growth chamber (24 °C/22 °C, 16 h/8 h daytime/nighttime cycles), that is, long-day conditions. (**C**) Image of the indicated alfalfa lines on Day 30 of the plants from (**B**). Bar = 3 cm. Branch number (**D**) and biomass (**E**) of the plants shown in (**C**). (**F**) Plant height of alfalfa plants grown outdoors in ‘environment II’, n = 4. Environment II: Outdoors from 19 September to 19 October 2023, Beijing (39°28′ to 41°03′ north latitude and 115°25′ to 117°35′ east longitude), namely short-day conditions. (**G**) Image of the indicated alfalfa lines in pots outdoors on Day 30. Bar = 3 cm. Branch number (**H**) and biomass (**I**) of the alfalfa plants shown in (**G**). Notes: The results were analyzed using a one-way ANOVA test. “*” represented *p* < 0.05, “**” for *p* < 0.01.

To characterize the biological function of *MsDREB1C* in alfalfa, *MsDREB1C* was overexpressed (OE), and two independent lines (OE2 and OE8) were analyzed in both hydroponic and soil-grown conditions. At the transcriptional level, both the OE2 and OE8 lines showed a significant increase of *MsDREB1C* in the seedlings compared to the non-transgenic alfalfa (Figure 4A). The effects of *MsDREB1C* overexpression on alfalfa growth were evaluated by planting the plants separately in hydroponics and soil for 30 days. The hydroponic plants were cultured in a growth chamber with a photoperiod of 16 h light/8 h dark and a temperature of 24 °C/22 °C (day/night). The plants in the pot were kept outdoors from 19 September to 19 October (which is when it is autumn in Haidian, Beijing) under short-day conditions.

Plant height (PH) was monitored every third day. For the first three measurements (Day 0–Day 6), no difference was observed between the two OE lines (OE2 and OE8) and WT. From Day 9, both transgenic lines were significantly shorter than WT, and PH from Day 21 to Day 30 displayed a significant difference between OE2 and OE8 (Figure 4B). On Day 30, the PH of OE2 and OE8 was about 18.85 cm and 21.4 cm, which is 7.9 cm and 5.3 cm shorter than WT, respectively. The node number of the main stem for both OE2 and OE8 was minorly smaller than WT (7.1 and 7.6 vs. 8.5), and the calculation of average internode length (AIL) revealed a decrease (Table S2). In addition, the branch number on average of the transgenic plants increased slightly (Figure 4D). Consequently, the biomass (fresh weight and dry weight) (Day 30) of OE2 but not OE8 was significantly lower than WT, and the ratio of dry weight to fresh weight (DFR) for both lines decreased significantly (Figure 4E and Table S2).

For the plants in the pot outdoors, the first four measurements (Day 0–Day 9) of PH were similar to the hydroponic ones in the growth chamber. Afterwards, the former plants displayed shorter PH, and PH on Day 30 was about half of the latter (i.e., WT: 12.1 cm vs. 26.7 cm; OE2: 8.6 cm vs. 18.9 cm; OE8: 9.7 cm vs. 21.4 cm) (Figure 4B,F). The results suggested that both the OE lines and WT grew slower in fall than under the long-day conditions with a high temperature of 24 °C/22 °C (day/night). Compared to WT, the PH of OE2 was significantly lower from Day 9, and OE8 from Day 15 (Figure 4F). These plants had similar node numbers in the main stem, resulting in a significant decrease of AIL in the *MsDREB1C* overexpression alfalfa (Table S2). The branch number of both OE lines increased (Figure 4H), and the biomass difference was not significant from that of wild-type alfalfa (Figure 4I).

### 2.5. Overexpression of MsDREB1C Reduced Gibberellin (GA) Synthesis

**Figure 5:**
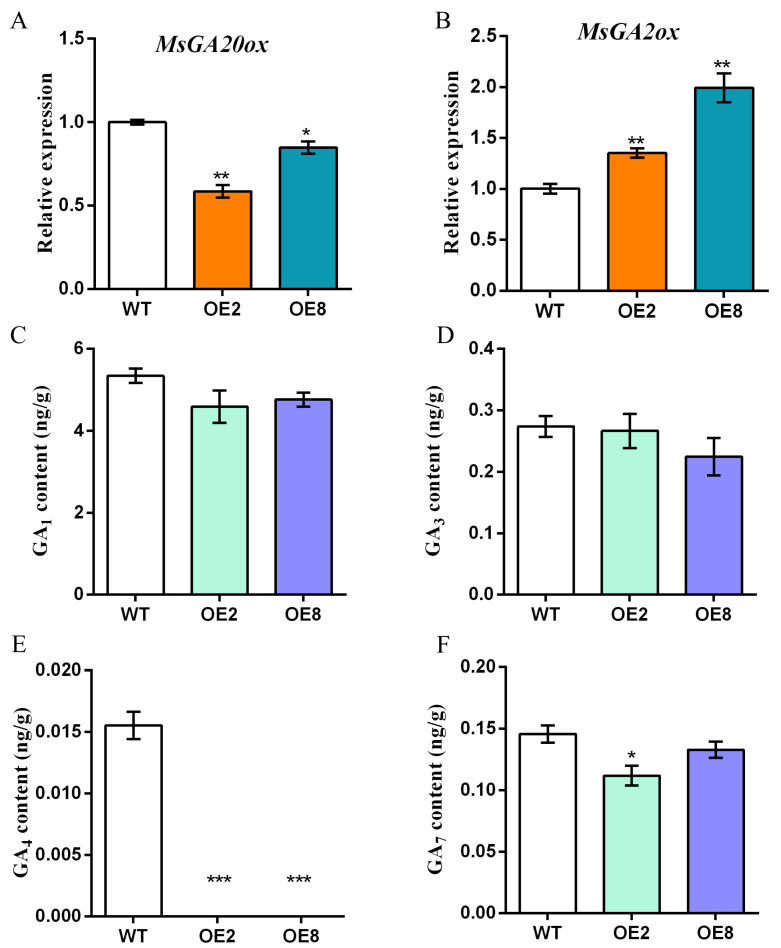
Overexpression of *MsDREB1C* in alfalfa affected GA synthesis. (**A**) Relative expression level of *MsGA20ox* and *MsGA2ox* (**B**) in the main stem of one-month-old soil-grown alfalfa. Measurement of (**C**) GA_1_, (**D**) GA_3_, (**E**) GA_4_, and (**F**) GA_7_ content in the main branch of one-month-old soil-grown alfalfa. Notes: Samples were taken from at least 4 independent plants. The results were analyzed using a one-way ANOVA test. “*” represents *p* < 0.05, “**” for *p* < 0.01. “***” for *p* < 0.001. Data are means ± SEM of three biological replicates.

As GA is an important phytohormone that regulates plant growth and development, we examined in alfalfa the expression of *Gibberellin 20 oxidase* (*MsGA20ox*) and *Gibberellin 2-oxidase* (*MsGA2ox*), key genes in GA biosynthesis and inactivation, respectively. The quantitative RT-PCR results showed that the transcription level of *MsGA20ox* in OE2 and OE8 was about 60% and 80% of that in WT, respectively (Figure 5A), while *MsGA2ox* in OE2 and OE8 was about 140% and 200% of the control, respectively (Figure 5B).

At the same time, we also detected the content of GA_1_, GA_3_, GA_4_, and GA_7_ with biological activity in GA. The results showed that the content of GA_1_ and GA_3_ in the OE lines was slightly lower than that in WT, although there was no significant difference compared with WT (Figure 5C,D), while GA_4_ was not detectable in the OE lines (Figure 5E). In addition, the content of GA_7_ also decreased in *MsDREB1C* overexpression lines, and the content of GA_7_ in OE2 and OE8 was about 73% and 87% of that in WT, respectively (Figure 5F). The findings suggest that overexpression of *MsDREB1C* has a negative effect on the accumulation of active GA in alfalfa, resulting in growth retardance of transgenic alfalfa.

### 2.6. Overexpression of MsDREB1C in Alfalfa Improves Forage Quality by Hindering Lignification in Stem

**Figure 6:**
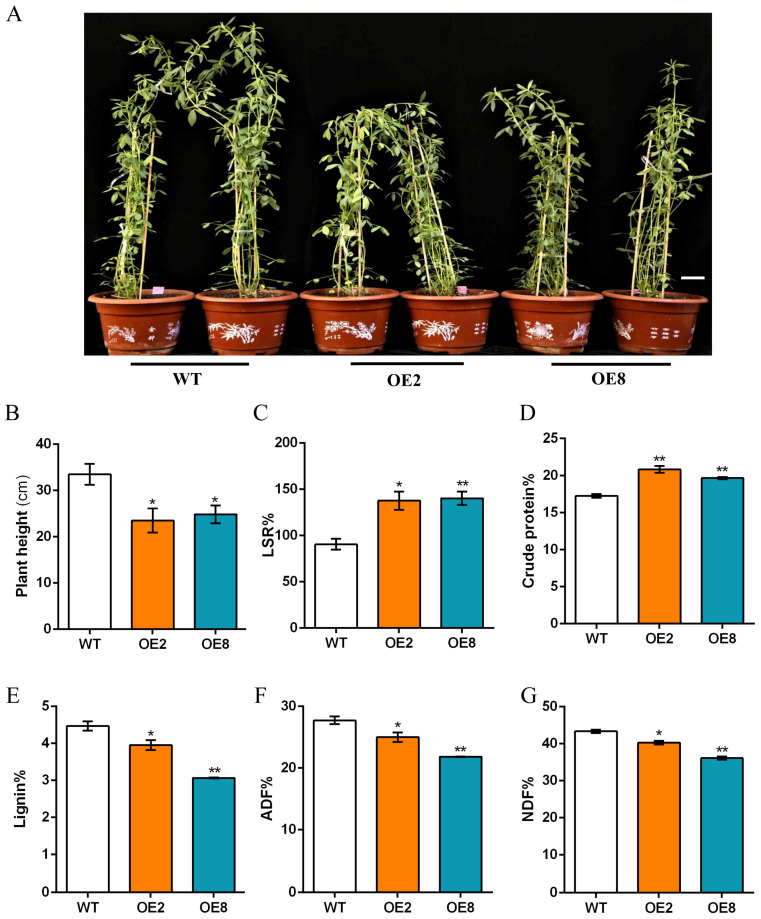
Overexpression of *MsDREB1C* improved alfalfa forage quality. (**A**) Image of the indicated alfalfa plants grown in a pot (17 cm diameter) for 6 weeks. Bar = 3 cm. (**B**) Plant height, (**C**) Leaf–stem ratio (LSR), (**D**) %crude protein, (**E**) %lignin, (**F**) %acid detergent fiber (ADF), and (**G**) %neutral detergent fiber (NDF) of the plants shown in (**A**). “*” represents *p* < 0.05, “**” represents *p* < 0.01. Note: The results were analyzed using a one-way ANOVA test. Data are means ± SEM of three biological replicates.

As the forage quality is determined by the leaf–stem ratio (LSR), we separated leaves from the stems of alfalfa grown in a pot for 6 weeks and measured the dry weight. The results showed that the LSR of the *MsDREB1C* overexpression lines was about 25–49% higher than that of WT (*p* < 0.05) for the first and second cuttings (Figure 6C and Figure S1A). Consistent with the previous experimental results, the plant height of the transgenic alfalfa after 6 weeks of planting in the greenhouse was also significantly lower than the wild type (Figure 6B).

Our evaluation of forage quality demonstrated that crude protein content in the *MsDREB1C* overexpression alfalfa was higher than that in WT for the first two clippings (Figure 6D and Figure S1B), and the content of lignin (Figure 6E and Figure S1C), acid detergent fiber (ADF) (Figure 6F and Figure S1D), and neutral detergent fiber (NDF) (Figure 6G and Figure S1E) in both OE lines decreased significantly. These results suggest that overexpression of *MsDREB1C* in alfalfa improves forage quality.

**Figure 7:**
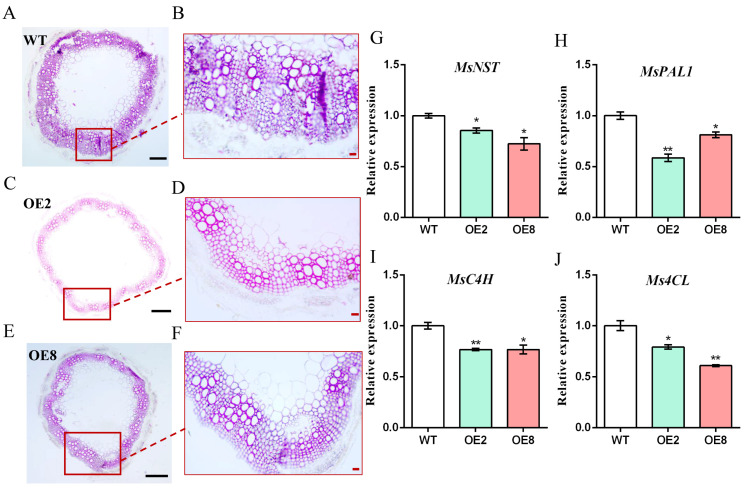
Histochemical staining of alfalfa stem. The tenth internode (from the top of the stem) from 6-week-old alfalfa was cross-sectioned and stained with m-phloroglucinol ethanol and HCl. The cross section of a stem from WT (**A**,**B**), Line 2 of the *MsDREB1C* overexpression alfalfa (**C**,**D**), and Line 8 of the *MsDREB1C* overexpression plants (**E**,**F**). The image was captured using an optical microscope (Olympus microscope CX-21, Japan). Scale bar = 20 μm for (**B**,**D**,**F**), and 200 μm for (**A**,**C**,**E**), respectively. Relative expression analysis of *MsNST* (**G**), *MsPAL1* (**H**), *MsC4H* (**I**), and *Ms4CL* (**J**). Stem from 6-week-old alfalfa was used for RNA isolation. Notes: Samples were taken from at least 4 independent plants. The results were analyzed using a one-way ANOVA test. “*” represents *p* < 0.05, “**” represents *p* < 0.01. Data are means ± SEM of three biological replicates.

To examine the lignification of the *MsDREB1C* overexpression alfalfa stem, histological analysis using the Wiesner test [12] was conducted. The tenth internode from the top of alfalfa at the 6-week stage was sectioned and dyed using phloroglucinol–HCl to detect the pink stain of lignifying xylem cell walls. As shown in Figure 7A–F, the degree of pink staining in stem tissues from OE2 and OE8 appeared weaker compared with that in the non-transgenic plant, indicating that overexpression of *MsDREB1C* weakens the extent of lignin present in the xylem. These results suggest that *MsDREB1C* might play a positive role in hampering the lignification of stem cell walls in alfalfa. In agreement with the findings of lignin visualization, the expression level of several key genes in lignin synthesis, including *NAC secondary-wall thickening factor (MsNST)*, *phenylalanine ammonia lyase1 (MsPAL1)*, *cinnamate-4-hydroxylase (MsC4H)*, and *4-coumarate:CoA ligase (Ms4CL)*, in both OE2 and OE8 was significantly lower than in WT (Figure 7G–J). Thus, overexpression of *MsDREB1C* negatively regulated the transcription of the lignin synthesis involving genes, thereby reducing lignin content in the transgenic plants, as indicated by the phloroglucinol–HCl reagent.

## 3. Discussion

Plant-specific transcription factor (TF) dehydration-responsive element-binding protein (DREB), a member of the APETALA2/ethylene-responsive element binding factor (AP2/ERF) family, has been well studied in response to abiotic stress in various plant species [14,15]. Recent studies have documented the involvement of *DREB* in plant growth and development, including photosynthesis, nitrogen use efficiency, and flowering [1,25]. It is tempting to speculate that *DREB* plays a vital role as an integrator of multiple biological processes, as well as plant defense against adverse environmental cues. Our observations of the pleiotropic effects, including growth retardation and improved hay quality of the *MsDREB1C* overexpression alfalfa, are supportive of the notion, suggesting that *MsDREB1C* may serve as a potential target for alfalfa breeding via genetic modification to balance multiple traits such as forage quality, plant growth, and resilience.

It appears that *MsDREB1C* plays a negative role in alfalfa growth, probably by transcriptional activating *gibberellin 2-oxidase* (*MsGA2ox*), which results in the deactivation of gibberellic acid (GA). An increasing body of evidence has shown that overexpression of DREB family members confers stress tolerance to a certain degree, but also leads to severe growth retardation due to disturbed GA signaling [23,25,26,27]. For example, overexpression of *DWARF AND DELAYED FLOWERING 1* (*DDF1*), a DREB1 protein in Arabidopsis, resulted in dwarfism and delayed flowering, together with increased expression of the *GA2ox* gene, which leads to reduction of GA biosynthesis [26]. Constitutive expression of soybean *DREB3* (*GmDREB3*) in Arabidopsis caused retarded growth, and the negative effect on plant growth could be minimized by driving the gene with the stress-inducible *Rd29A* promoter [27]. Research on *M. truncatula*, the closest model legume of alfalfa, has revealed enhanced freezing tolerance and suppressed plant growth of the *MtDREB1C* overexpression *M. truncatula* [20]. Interestingly, overexpression of maize *ZmDREB4.1*, which was uninducible by abiotic treatment, in tobacco caused repression of leaf extension and elongation of hypocotyl, petiole, and stem, and GA reduction was observed in most of the organs [25]. In agreement with these findings, we found that overexpression of *MsDREB1C*, an abiotic inducible gene encoding a nuclear protein with self-transactivation activity, in alfalfa led to discernably shorter plant cultured either hydroponically under long-day conditions or in a pot outdoors under autumn short-day conditions. The abnormal phenotype of the OE lines is reminiscent of GA-insufficient mutants [28]. Indeed, in the transgenic lines, the expression level of *MsGA2ox* was significantly elevated (*p* < 0.01), and *MsGA20ox*, a key gene in GA_1_ and GA_4_ biosynthesis, decreased (*p* < 0.05) relative to the non-transgenic plants. Exogenous application of GA_3_ to the dwarf transgenic alfalfa would provide evidence of the alteration of active GA level caused by *MsDREB1* upregulation. In addition, tomato *SlDREB* has been documented to restrict leaf expansion and internode elongation by downregulating *GA 20-oxidases* [21,29]. On the other hand, a *C-REPEAT BINDING FACTOR* in tomato (*SlCBF*) was activated by PHYTOCHROME-INTERACTING TRANSCRIPTION 4 (SlPIF4), which could directly activate *SlDELLA* under cold treatment, implying the involvement of GA signaling [21]. Moreover, GA_1_, GA_3_, GA_4_, and GA_7_ are four gibberellins reported to have high biological activity in angiosperms [30]. In our study, after overexpression of *MsDREB1C* in tetraploid alfalfa, different degrees of decrease in these four gibberellins were observed (Figure 5). A plausible explanation for the observations is that growth reduction allows resources to be concentrated preferentially on withstanding the stress plants face, which is facilitated by the homeostasis of phytohormone GA. Considering the growth/yield reduction caused by constitutive expression of stress-related regulatory genes, stress-inducible promoters have been applied to optimize the expression levels of transgenes and proven to be effective in improving plant developmental phenotypes and yield [31]. It would be informative to investigate the biological functions of *MsDREB1C*’s paralogs, particularly the four tandemly arranged ones flanking *MsDREB1C* on chromosome 6, in alfalfa despite no clear expression alteration by short-term (24 h) cold stress.

The *MsDREB1C* overexpression alfalfa seems to deliver competitive levels of forage quality. As an excellent source of high-quality protein, as well as fiber, alfalfa is a staple ingredient in most dairy cow diets around the globe [2]. Forage yield and nutrient content are usually evaluated based on dry matter, including the non-structural parts of the plant tissue, such as protein, sugar, and starch, and the structural components, i.e., cellulose, hemicellulose, and lignin. In alfalfa, the content of crude protein and cellulose (ADF, NDF, lignin) are the main evaluation indicators of forage quality [7]. Our soil-cultured *MsDREB1C* overexpression alfalfa did not differ in biomass from that of the non-transgenic control, which is likely attributed to the increase of branch numbers. Owing to the short plant stature, the leaf–stem ratio for the overexpression lines was about 25–49% higher than for the control plants. As a result, the crude protein content increased (1.6–3.7%). It is unclear how *MsDREB1C* contributes to the alteration of protein level in transgenic alfalfa. A recent study on *OsDREB1C* revealed that the allocation of both carbon and nitrogen from source to sink was more efficient in the *OsDREB1C* overexpression rice due to improved photosynthesis and nitrogen utilization, respectively, which led to an elevation of grain yield in rice and wheat [1]. For the lignin content, a reduction of 0.5–1.9% was detected in the *MsDREB1C* overexpression alfalfa compared with the control. Also, a mild repression of lignin biosynthesis involving genes, such as *MsNST*, *MsC4H*, *Ms4CL*, and *MsPAL1*, was monitored in the alfalfa OE lines. According to the transcriptomic data of the transgenic lines ubiquitously expressing *BaDBL1*, a DREB-encoding gene from moss (*Bryum argenteum*), in Arabidopsis, almost all tested lignin biosynthesis-related genes were not significantly different from WT under normal conditions, but most of them were upregulated by osmotic stress in the *BaDBL1* overexpression lines compared with Col-0 [32]. In contrast, the ectopic expression of *DcDREB1A* from wild carrot (*Daucus carota*) enhanced lignin accumulation in Arabidopsis leaves and stems with the upregulation of some lignin biosynthesis genes, including *AtPAL1*, *AtC4H*, and *At4CL1* in both tissues [33]. The composition of lignin may account for the discrepancy due to the fact that the complex composition and structures of these phenylpropanoid polymers vary with plant species and growth conditions [34]. In addition, the forage analysis provided a significantly lower measurement of both ADF (2.7–7.5%) and NDF (7.3–9.5%) in the *MsDREB1C* overexpression plants (*p* < 0.05), suggesting better fiber digestibility. These findings strengthen the hypothesis that *MsDREB1C* has an impact on lignin homeostasis in the alfalfa cell wall.

## 4. Materials and Methods

### 4.1. Plant Material and Growth Conditions

The alfalfa cultivar *Medicago sativa* L. cv. Zhongmu-1 was used as wild type in this study. For the hydroponic culture, seeds germinated on petri dishes were transferred to 1/2 Hoagland solution at a growth chamber with 24 °C/22 °C, 16 h/8 h daytime/nighttime cycles, 65 mmol m^−2^ s^−1^ light intensity, and 60–70% ambient humidity. Alfalfa with 4-week-old plants in hydroponic culture was used for tissue differential expression analysis and stress-related temporal expression measurement. The plants were cultivated separately in 1/2 Hoagland solution or 1/2 Hoagland solution with 200 mM NaCl or 15% mannitol or 4 °C. Leaf tissues from the NaCl and 4 °C treatment were collected at the time point of 0, 5, 12, and 24 h, and for mannitol treatment at 0, 8, 12, and 24 h, and leaves from the non-treated plants harvested at the corresponding time point were used as control. The transgenic lines obtained by genetic transformation in this paper were from one alfalfa (i.e., the wild type mentioned in this paper) and the alfalfa obtained by asexual reproduction of this alfalfa to ensure that the genotype differences between different plants were excluded. The wild-type alfalfa grown in soil culture for 4 weeks after cutting in the greenhouse was used to detect the expression of five *DREBs* after 4 °C cold treatment for 72 h, and the growth conditions in the greenhouse were basically the same as those in the growth chamber, with 24 °C/22 °C, 16 h/8 h daytime/nighttime cycles. For growth-related indicator experiments, all transgenic lines and wild-type plants were selected from simultaneous asexual reproduction with uniform-size and consistent growth conditions. Alfalfa grew for 30 days under two environmental conditions. ‘Environment I’ represents the conditions in the growth chamber mentioned above, that is, long-day conditions. ‘Environment II’ represents outdoor autumn conditions in Haidian, Beijing (39°28′ to 41°03′ north latitude and 115°25′ to 117°35′ east longitude), namely short-day conditions. Among them, the alfalfa grown in ‘environment I’ was hydroponic, and the alfalfa grown in ‘environment II’ was soil-cultured in pots. The height of alfalfa grown in the two environments was measured from 19 September to 19 October 2023. Additionally, six-week-old alfalfa grown in large pots (17 cm in diameter) with soil culture in greenhouse was used to measure the leaf–stem ratio, lignin content, and other forage quality-related indicators.

### 4.2. Total RNA Isolation, Reverse Transcription, and Quantitative RT-PCR Analysis

Total RNA was extracted using the Promega Eastep™ Total RNA Extraction Kit (Promega, Beijing, China) according to the manufacturer’s instructions. The RNA concentrations were detected using NanoPhotometer™ Microvolume Spectrophotometer (IMPLEN, Beijing, China). First-strand cDNA synthesis was performed using the UnionScript First-strand cDNA Synthesis Mix for qPCR Kit (Jinsha, Beijing, China) following the manufacturer’s instructions. Quantitative RT-PCR analysis was performed according to the manufacturer’s instructions for an aq Pro Universal SYBR qPCR Master Mix Kit (Vazyme, Nanjing, China) using an ABI 7300 Real-Time system (Bio-Rad, Hercules, CA, USA).

### 4.3. Plasmid Construction

The full-length coding sequence of *MsDREB1C* was amplified using the primers MsDREB1C-F and MsDREB1C-R (Table S3). The *MsDREB1C* overexpression plasmid was generated using the SmaI restriction site of Super1300 expression vector preserved in our laboratory. The sequence verified construct was transferred into the *Agrobacterium* EHA105 strain (Zoman, Beijing, China). For generation of the *Super promotor:MsDREB1C-GFP* fused construct, the coding sequence of *MsDREB1C* without the stop codon was amplified by PCR using the primers MsDREB1C-1300-F and MsDREB1C-1300-R (Table S3), and constructed into Super1300 expression vector using the Sma Ⅰ restriction site. Sequence-verified plasmid and the GFP control vector were transformed into the *Agrobacterium* GV3101 strain (Zoman, Beijing, China), separately. For the construction of BD-MsDREB1C, the coding sequence of *MsDREB1C* was first amplified by the primers MsDREB1C-BD-F and MsDREB1C-BD-R (Table S3), and the correct sequence was then connected to the pGBKT7(BD) vector by seamless cloning enzyme to obtain the BD-MsDREB1C fusion vector.

### 4.4. Bioinformatic Analysis

DNAMAN was used for sequence analysis and sequence alignment, and EXPASY (https://web.expasy.org/compute_pi/, accessed on 8 March 2023) for protein features of molecular weight and the isoelectric point. The phylogenetic tree was constructed using MEGA (v11.0, Tamura, K., Tokyo, Japan) and iTOL (https://itol.embl.de/, accessed on 12 June 2023).

### 4.5. Plant Transformation and Infiltration

The genetic transformation of alfalfa was performed following the protocol [35] with hygromycin B of 5 mg/L for preliminary selection. The transgenic plants were confirmed by PCR using the primers 1300-F and MsDREB1C-1300-R (Table S3). For protein localization, the *Agrobacterium* GV3101 carrying the *MsDREB1C-GFP* recombinant protein or GFP was infiltrated into tobacco (*Nicotiana benthamiana*) leaves. After 48 h culture, the epidermal cells of the transformed tobacco were imaged under a confocal microscope (Leica, TCS SP8, Wetzlar, Germany).

### 4.6. Self-Transactivation Assay in Yeast

The plasmids of BD-MsDREB1C and pGBADT7 (AD) were co-transformed into Y2H yeast cells. Yeast cells harboring BD-MsDREB1C and AD co-transformation product were grown on two media. I. SD/-Trp, -Leu (DDO) medium with X-α-gal; and II: SD/-Trp, -Leu, -His, -Ade (QDO) medium with X-α-gal. The BD-53 and AD-T co-transformation product was used as positive control, the BD-lam and AD-T co-transformation product as negative control, and the BD and AD co-transformation product as double negative control.

### 4.7. Measurement of Growth-Related Indicators

For the growth indicators, the plants were measured under long-day (‘environment I’) and in pots outdoors under autumn short-day (‘environment II’) conditions. The plants were initially pruned to the same stubble height. Plant height was measured from the ground to the top point of the longest branch with a ruler in centimeters (cm). The measurement was performed every 3 days until Day 30. Branch number, node number on main stem, and fresh weight were measured on Day 30, and the samples were dried at 65 °C for two days for the dry weight. For calculation,

Average internode length of main stem (cm) = Plant height/node number on main stem

DFR = Dry weight/Fresh weight

### 4.8. Measurement of Gibberellin (GA) Content

The main branches of *MsDREB1C* overexpression and WT alfalfa plants cultured in soil for one month were collected and immediately frozen in liquid nitrogen. The content of GA was detected using ultra-performance liquid chromatography–tandem mass spectrometry (UPLC-MS/MS) according to a previous method described in [24].

### 4.9. Lignification Analysis

Fresh samples of the tenth stem segments, counting from the top of the 6-week-old plant, were cross-sectioned into 20–25 μm thin slices and stained with 2% phloroglucinol staining solution after dewaxing and hydration according to a previous method [12]. The sealed sample was imaged under an optical microscope (Olympus microscope CX-21, Tokyo, Japan).

### 4.10. Forage Quality Measurement

For quality evaluation, the aboveground tissues were harvested, leaving 5 cm of stubble. The stems and leaves were separated for dry weight. Dry leaf weight (DLW), dry stem weight (DSW), crude protein, lignin, acid detergent fiber (ADF), and neutral detergent fiber (NDF) were measured after defoliating at 105 °C for 25 minutes, and subsequent oven-drying at 65 °C for two days. The content of lignin, ADF, NDF, and crude protein was determined using near-infrared reflectance spectroscopy (Foss NIRS DS 2500 F, Denmark). LSR was calculated using the following equations:

Leaf/stem ratio (LSR) = DLW/DSW × 100%

### 4.11. Statistical Analysis

The raw data were collated in Microsoft Excel (2016, Microsoft, Redmond, WA, USA), and SPSS 22.0 (SPSS Statistics, Chicago, IL, USA, IBM 22.0) was used for one-way ANOVA analysis and Duncan’s multiple comparison. Image drawing and image integration were performed in Prism 6.01 (GraphPad Software Inc., San Diego, CA, USA).

## 5. Conclusions

**Figure 8:**
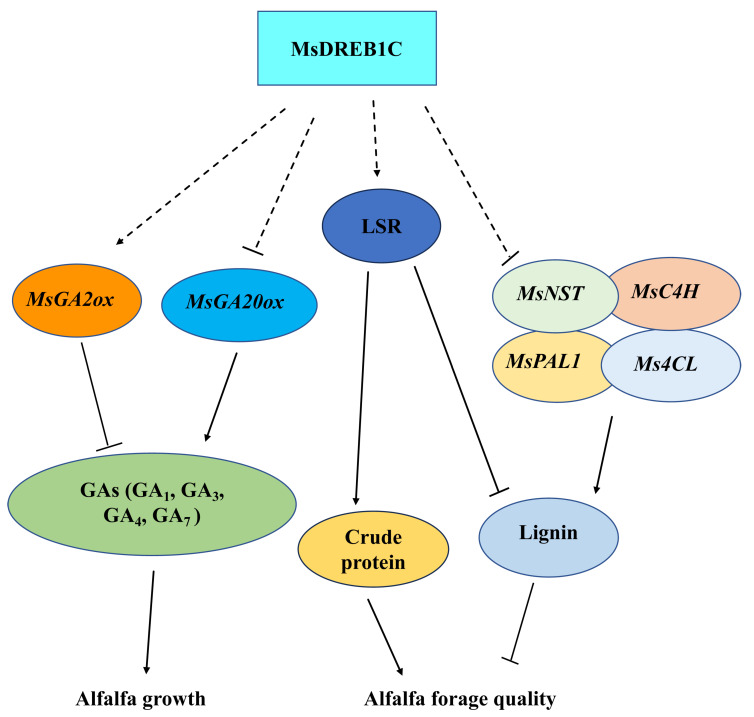
The possible regulatory mechanism of *MsDREB1C* in regulating the growth and quality of alfalfa. The overexpression of *MsDREB1C* in alfalfa enhances the transcription of *MsGA2ox* and decreases *MsGA20ox* expression level, which results in the reduction of gibberellin (GA_1_, GA_3_, GA_4_, GA_7_) synthesis. On the other hand, *MsDREB1C* increases crude protein and decreases lignin content by boosting the leaf–stem ratio and suppressing the expression of genes related to lignin synthesis, such as *MsNST*, *MsC4H*, *MsPAL1*, and *Ms4CL*, to improve forage quality.

This study identified the pleiotropic effects of the abiotic inducible gene *DREB1C* on delaying growth and improving forage quality in perennial tetraploid alfalfa, and indicated that this effect was closely related to GA synthesis (GA_1_, GA_3_, GA_4_, GA_7_) and lignin synthesis (Figure 8). In future, the prospect of *MsDREB1C* as a potential candidate for the improvement of growth, forage quality, and resilience in alfalfa would be promising because of its highly conserved functional domain and diverse target genes, as well as the high feasibility of expression alteration via genetic engineering.

## Data Availability

Data are contained within the article and Supplementary Materials.

## Supplementary Materials

The following supporting information can be downloaded at: https://www.mdpi.com/article/10.3390/plants13091237/s1, Figure S1: Determination of forage quality of alfalfa after second clipping; Table S1: The physical positions of 5 *DREB* genes on chromosome; Table S2: Comparison of growth-related indicators in *MsDREB1C*-overexpressing and wild type alfalfa; Table S3: Primers sets used in this study.
