# Supplementary material for: Overexpression of MsDREB1C Modulates Growth and Improves Forage Quality in Tetraploid Alfalfa (Medicago sativa L.)
Source: Plants (Basel). 2024 Apr 29;13(9):1237. doi: 10.3390/plants13091237 (PMC11085332; doi:10.3390/plants13091237)
Supplement: Supplementary file 1 [file plants-13-01237-s001.zip › plants-2945873-supplementary.pdf]

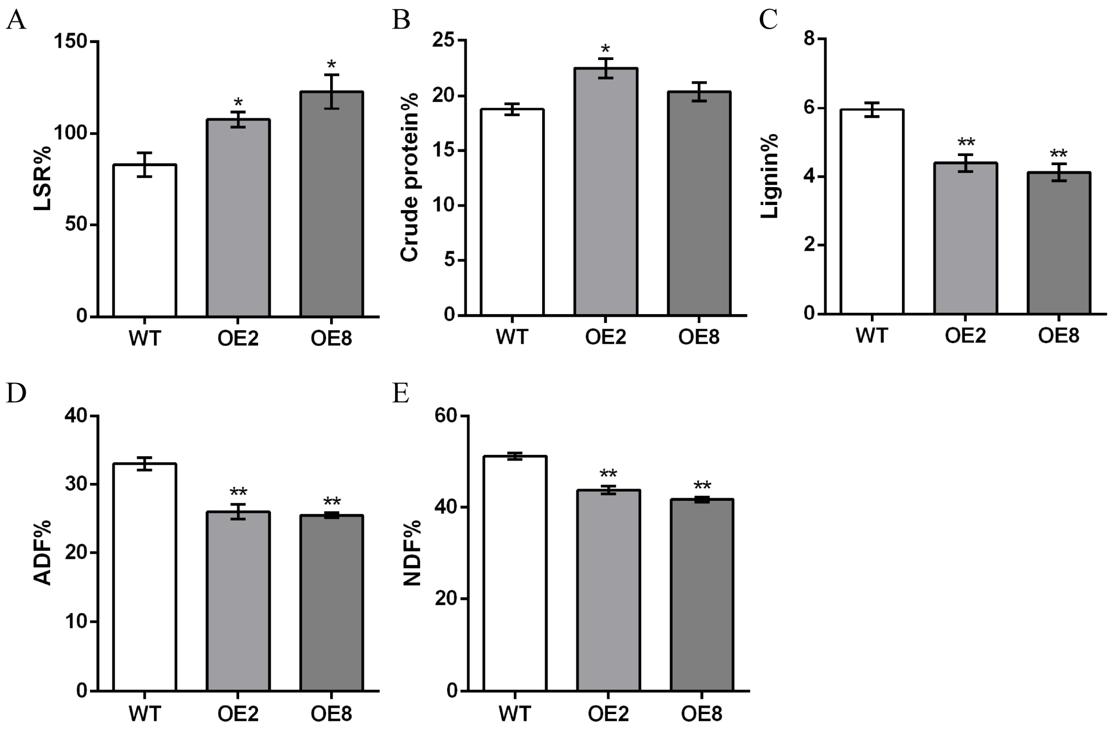

Figure S1 Determination of forage quality of alfalfa after second clipping. Leaf-stem ratio (LSR) (**A**), % crude protein (**B**), % lignin (**C**), % acid detergent fiber (ADF) (**D**), % neutral detergent fiber (NDF) (**E**) in both WT and transgenic plants (OE2, OE8). "*" represents p<0.05, "**" represents p<0.01. Note: The results were analyzed using a one-way ANOVA test. Data are means ± SEM of three biological replicates.

Table S1 The physical positions of 5 DREB genes on chromosome

Gene Name	Chromosome	Start position	Stop position	Direction on the
Msa0891950	Chr6	69234712	69239739	Forward
Msa0891960	Chr6	69344692	69345384	Forward
Msa0891970	Chr6	69357058	69357738	Forward
Msa0891980	Chr6	69374816	69375502	Forward
Msa0891990	Chr6	69523664	69525980	Forward

Table S2 Comparison of growth-related indicators in *MsDREB1C*-overexpressing and wild type alfalfa

	Environment I			Environment II		
	MNN	AIL(cm)	DFR	MNN	AIL(cm)	DFR
WT	8.5	3.1	0.31	5.3	2.4	0.21
OE2	7.1 *	2.6 *	0.28 *	5.3	1.7 *	0.19
OE8	7.6	2.8	0.27 *	6	1.6 *	0.23 **

Note: "MNN" represents numbers of node on main stem. "AIL" represents average internode length of main stem (Height / MNN). "DFR" represents the ratio of dry weight to fresh weight. All indicators are measured after 30 days of plant growth. 'Environment I' represents the conditions in the growth chamber (24 °C/22 °C, 16 h/8 h daytime/nighttime cycles), that is, long-day conditions. 'Environment II' represents outdoor autumn conditions in Haidian district, Beijing, namely short-day conditions. Among them, the alfalfa grown in 'environment I' was hydroponic, and the alfalfa grown in 'environment II' was soil-cultured in pots. The growth height of alfalfa grown in two environments was measured from September 19 to October 19. Note: The results were analyzed using a one-way ANOVA test. "*" represents p<0.05, "**" represents p<0.01.

Table S3 Primers sets used in this study

Primer	Sequence (5′-3′)
Ms0891950-RT-F	GGAACAAAGACATATGTGTCAGGA
Ms0891950-RT-R	AGTTGAGACAGGCGTAACGG
Ms0891960-RT-F	GTAGGACTTGTATGGAAGAGGAA
Ms0891960-RT-R	ACCTCTTCATCTTGAAAATCTGGA
Ms0891970-RT-F	GGCTATTGCCACTACGGAGG
Ms0891970-RT-R	TGTGTAGGAGACATTAGCGCC
Ms0891980-RT-F	CTAGGCACTTTTCCGACAGC
Ms0891980-RT-R	GTCTTGTCCGGTCTGAAAGC
Ms0891990-RT-F	CGACTATAGCCGCCTCTACC
Ms0891990-RT-R	TGGCATGTTCATCGATCCCT
MsDREB1C-F	ATGGATATGTTAACAACTAACAACTC
MsDREB1C-R	TTAATAATTCCATAGTGATACCTCTTC
MsDREB1C-1300-F	TCTAGAAAGCTTCTGCAGGGGATGGATATGTTAACAACTAACAA CTC
MsDREB1C-1300-R	CTCCTCGCCCTTGCTCACCATATAATTCCATAGTGATACCTCTTC
1300-F	ACAACGCTTTACAGCAAGAACGG
1300-R	GGTCAGGGTGGTCACGAGGG
MsDREB1C-BD-F	CATGGAGGCCGAATTCATGGATATGTTAACAACTAA CAAC
MsDREB1C-BD-R	GCAGGTCGACGGATCCTTAATAATTCCATAGTGATA CCTC
MsActin-F	CAAAAGATGGCAGATGCTGAGGAT

MsActin-R	CATGACACCAGTATGACGAGGTCG
MsDREB1C-RT-F	CTAGGCACTTTTCCGACAGC
MsDREB1C-RT-R	GTCTTGTCCGGTCTGAAAGC
MsNST-RT-F	TGGCTTGCTTTAATGACCCC
MsNST-RT-R	TCGAGCAAAGTTCCAGAGGT
MsPAL1-RT-F	CGGGTTTGTTAACCGGCAGA
MsPAL1-RT-R	ACTTCAGCGAAAATTGCCGAC
MsC4H-RT-F	GAAGTCCTCCACACACAAGGT
MsC4H-RT-R	CAACACTCGCTTCAGCGTT
Ms4CL-RT-F	CCCGAGTTCCACTCACTCAC
Ms4CL-RT-R	CTGTCAGCAAACAAACCGCA
MsGA20ox-RT-F	GAATGAGCCTTGGAGTTGG
MsGA20ox-RT-R	TGGAATGCCATTGATCGTC
MsGA2ox-RT-F	TCCCCTGCTTTTGGTAAAGAT
MsGA2ox-RT-R	GGACAAGGGGGATAATGGTTG
